# Naïve Rat NK Cells Control the Onset of T Cell Response

**DOI:** 10.1371/journal.pone.0047074

**Published:** 2012-10-15

**Authors:** Lilli Kraus, Britta Trautewig, Juergen Klempnauer, Thorsten Lieke

**Affiliations:** 1 Transplant Laboratory, Department of General-, Visceral- and Transplantation Surgery, Hannover Medical School, Hannover, Germany; 2 ReMediES, Department of General-, Visceral- and Transplantation Surgery, Hannover Medical School, Hannover, Germany; National Jewish Health and University of Colorado School of Medicine, United States of America

## Abstract

NK cell function in the rat is only defined in a rudimentary way due to missing tools for clear NK cell identification. The present study introduces the congenic LEW.BH-NKC rat strain which allows distinct detection of rat NK cells using commercial antibodies. LEW.BH-NKC rats were exposed *in vivo* to the porcine B cell line L23 by subcutaneous transfer of L23 cell suspension. We used Luciferase transgeneic L23 cells to follow the course of rejection by living imaging. L23 cells were rejected within five days after placement under the skin thus the rejection is mediated by innate immune responses in the first place. Indeed we found increased percentages of NK cells in the blood, spleen and in draining lymph nodes using flow cytometry methods. Surprisingly, we found as a consequence a decrease in proliferative T cell response in the draining lymph nodes. We identified NK cells as mediators of this regulation by *in vitro* performed mixed lymphocyte reactions. The remarkable feature was the naive state of NK cells exhibiting the regulative capacity. Furthermore, the regulation was not exclusively mediated by IL-10 as it has been reported before for influence of T cell response by activated NK cells but predominantly by TGF-β. Interestingly, after initiation of the adaptive immune response, NK cells failed to take influence on the proliferation of T cells. We conclude that naive NK cells build up a threshold of activation impulse that T cells have to overcome.

## Introduction

Natural killer (NK) cells were identified in the 1970 as a non-phagocytic lymphocyte population with high cytotoxic potential which at first exclusively was correlated with antibody dependent cytotoxicity (ADCC) [1], [2]. NK cells are assigned to the innate immunity building up a first line of defence following invasion of pathogens or transplantation of grafts by responding to the provocation within hours. Although cytotoxicity is a main feature of NK cells, a broad repertoire of cytokines are secreted depending on the stimulation of NK cells [3].

Due to the immediate response after direct engagement of activatory receptors, control of NK activation is crucial to prevent autoimmune response. This is guaranteed by constitutively expressed inhibitory receptors. In humans these receptors are assigned to the killer cell Ig-like receptors (KIR) and in rodents to the family of killer cell lectin-like receptors (KLR). The majority of these receptors interact with self-MHC I molecules and reveal cytoplasmatic immuno receptors tyrosine-based inhibitory motifs (ITIM) thus cells lacking self-MHC molecules are recognised as targets [4].

KLR predominantly includes the receptor families of Ly49 and NKR-P1 receptors [5]. In human only one KLR member namely NKR-P1A (CD161) can be identified whereas rodents express a variety of activatory and inhibitory NKR-P1 receptors [6]. In the rat, expression of a certain repertoire of different KLR has led to characterisation of functionally different cell subsets such as for the expression of Ly49 and NKR-P1C molecules on NK cells [7], [8], [9]. Functionally distinct subset of NK cells have also been found in human and mice but here classified by CD56 and CD16 in human [3] and CD27 in mice [10]. However, the identification of functional comparable NK subsets in different species does not correlate with comparable knowledge of NK cell biology. While a more and more detailed picture of NK cell function can be plotted in humans and mice, the data of NK cells in rats are still full of gaps. This is biased by the fact that rats – in contrast to mice – are resistant of germline modification using molecular techniques to generate transgenic or knock-out strains which resulted in alleviated popularity of rats as an animal model. This becomes obvious by reviewing commercially available antibodies against cells of the rat immune system. Commonly mAb 3.2.3 and 10/78 are used to identify NK cells. They are described to bind to NKR-P1A, a receptor expressed on all NK cells. However, with cross-reactivity to the inhibitory NKR-P1B receptor which is expressed on NK cells and monocytes thus staining of bulk lymphocyte population results in undetermined detection of NK cells [11].

Nevertheless, there are few indications that rat NK cells take part in the immune response after allogeneic [12] and xenogenic transplantation [13] and in infection with *Listeria monocytogenis* [14] and rat cytomegalovirus [15].

In this study we introduce a congenic rat strain lacking the allele of NKR-P1B recognised by mAb 3.2.3 and 10/78 thus these rats allow assessment of reactivity solely of NK cells. To follow the NK response we chose a xenogenic transplantation model by subcutaneous transfer of a cell suspension of the porcine B cell line L23 in the ear.

We found fast rejection of the L23 cells with a concurrent increase of NK cells and monocytes in the draining lymph nodes, spleen and in high frequency in the blood three days after cell transfer. Furthermore, we observed that invasion of NK cells in the draining lymph nodes can lead to control of T cell responses which sheds light of a new aspect of NK biology not only in rats but in general terms.

## Results

### Distinct Identification of NK Cells in the Congenic Rat Strain LEW.BH-NKC

**Figure 1:**
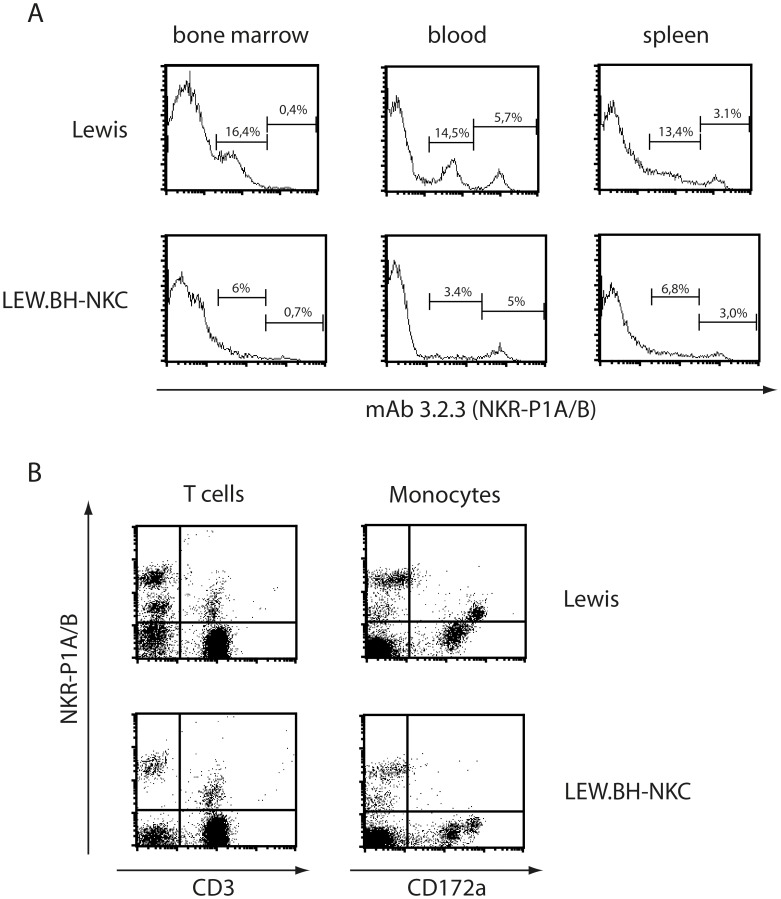
In congenic LEW.BH-NKC the monoclonal antibody 3.2.3 binds to NKR-P1A but not NKR-P1B molecules and allows clear identification of NK cells. A: Staining of Lewis wt and congenic LEW.BH-NKC bone marrow, blood derived lymphocytes and splenocytes with mAb 3.2.3 led to different recognition pattern in flow cytometry. While cells from Lewis wt showed two peaks indicating the detection two cell populations 3.2.3^high^ and 3.2.3^low^, congenic LEW.BH-NKC derived cells revealed a comparable percentage of 3.2.3^high^ cells but an extenuated peak of 3.2.3^low^ cells. **B:** Double staining of mAb 3.2.3 with anti-CD3 and anti-CD172a, a marker for monocytes, respectively. While the 3.2.3^high^ expressing cells remain single positive the cells expressing 3.2.3^low^ were either double positive for CD3 thus can be designated as NKT cells or expressed on CD172a^+^ monocytes. The 3.2.3^+^/CD3^+^ could be also detected in LEW.BH-NKC rats whereas the CD172a^+^ double positive cells were missing. These data indicate that the 3.2.3^high^ cells are NKR-P1A^+^ NK cells and the 3.2.3^low^ cells comprise NKR-P1B expressing monocytes or NKT cells. Results are shown as representative data of a typical screen of Lewis wt and LEW.BH-NKC rats.

The staining of cells derived from Lewis wt rats using mAb 3.2.3 does not give specific information about NK cells since this common antibody cross-reacts with NKR-P1B which is also expressed on monocytes **(** **Fig. 1B** **)**. Recently, a congenic rat strain was generated in our lab carrying the natural killer complex (NKC) of Black Hooded rats on Lewis rat background (from here designated as LEW.BH-NKC). The NKC of Black Hooded rats lacks the allele on NKR-P1B receptor recognised by mAb 3.2.3 thus only NKR-P1A is stained with these antibodies in LEW.BH-NKC **(** **Fig. 1A** **)**. Nevertheless, even NKR-P1A is not exclusively expressed on NK cells but also on NKR-P1A^+^/CD3^+^ NKT cells of the rat **(** **Fig. 1B** **)**. However, the diminished NKR-P1A expression on NKT cells allows discrimination of NK and NKT cells applying flow cytometry and negative selection ensures a pure NK cell isolation. Thus, all following experiments were performed using LEW.BH-NKC congenic rats and negative isolated NK cells.

### Porcine B Cell Grafts are Quickly Rejected and Induce NK Mediated Cytotoxicity but Decrease the Proliferation of Draining Lymph Nodes

**Figure 2:**
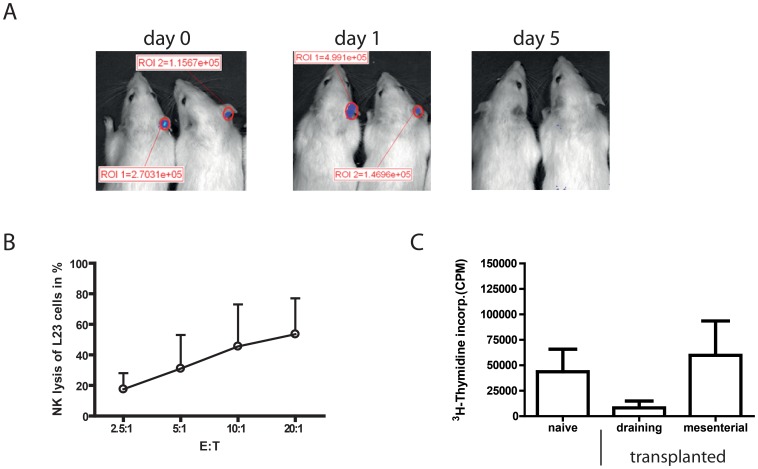
Subcutaneous transplantation of L23 cells in the ear induce fast rejection and reduced proliferative response of draining lymph node cells. A: Living imaging of Luciferase-transgenic porcine B cell line L23 on day 0, 1 and 5 after placing 4×10^5^ cells subcutaneous in the ear of Lewis wt rats. Luminescence intensity is given in p/sec/cm^2^/sr indicated as ROI. Screen of viable L23 cells revealed a fast rejection of porcine cells within 5 days. **B:** *In vitro* cytotoxicity assay of naive NK cells against ^3^H thymidine labelled L23 in different effector (E) to target (T) ratios using the JAM test. The assay revealed pronounced lysis of L23 cells. Cytolytic activity is a possible explanation for the fast rejection of the graft and an indication direct activation of naive NK cells by L23 cells. **C:** Preparation of cervical lymph nodes of naive Lewis wt rats and draining (cervical) and mesenterial lymph nodes of transplanted Lewis wt rats 3 days post transplantation, respectively. Lymphocytes were (re-)stimulated with 2×10^3^ irradiated L23 cells for 5 days. Comparison of proliferative response showed a decrease of ^3^H thymidin incorporation in draining lymph nodes. Results are shown as representative data or as graphs summarising results of at least three independent experiments as mean ± standard deviation.

We established a xenogenic transplantation model by placing Luciferase-transgenic L23 cells, a porcine B cell line, under the skin of Lewis wt and LEW.BH-NKC congenic rats. L23 cells were used as they have known capacity to activate immune responses directly [16]. The course of rejection was followed using IVIS imaging. On day 1 after transplantation, L23 cells were well detectable in the ear of Lewis wt rats whereas on day 5 none of the graft cells remained **(** **Fig. 2A** **)**. This fast rejection makes the model interesting to evaluate NK cell participation during the process of rejection because this has to be mediated mainly by innate immunity.

One of NK cell major characteristics is the fast and direct lysis of targets expressing a different MHC haplotype. Thus, it is possible that NK cells attack the porcine L23 cells and destroy them. Indeed, cytotoxicity assay with L23 as targets and negative isolated, naive NK cells as effectors revealed a pronounced lysis of L23 cells **(** **Fig. 2B** **)**. Hence, a reasonable explanation of the fast rejection would be a clearance of the porcine cells solely by NK cells without any induction of adaptive immunity.

In actual fact, the missing adaptive response ought to be confirmed by comparison of response of lymphocytes derived from draining and mesenteric lymph node from the same individual. Before specific T cell activation the proliferative response should be similar after stimulation with L23 cells *in vitro*. Therefore, rats were sacrificed at day 3 after transplantation as an intermediate time point at which some rats revealed remaining L23 cells whereas other have rejected the transplants (data not shown). To our surprise we obtained contradictive results: a clear however not significant decrease of proliferation in draining lymph nodes **(** **Fig. 2C** **)**.

This gives an indication that in addition to fast activation of NK cells leading assumingly to rejection of grafts, regulative processes prevent an early activation of an adaptive T cell response.

### Transplantation of Porcine B Cells Leads to Increased Numbers of NK Cells in Draining Lymph Nodes

**Figure 3:**
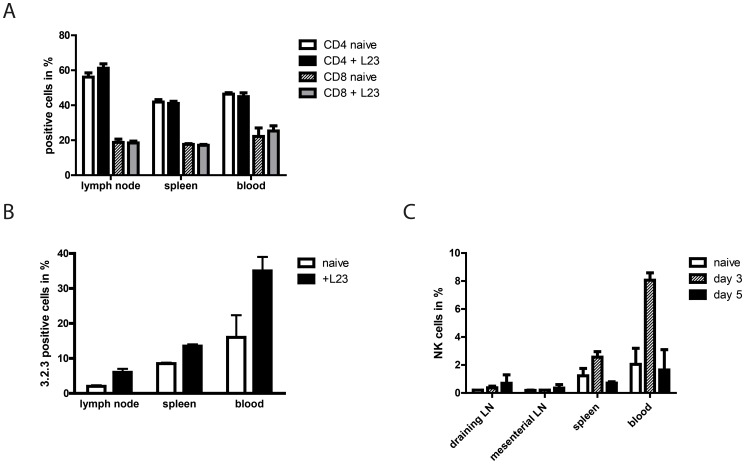
NK cells infiltrate the draining lymph nodes and expanded proportion in spleen and blood in the early phase of immune response. A: Analysis of CD4^+^ and CD8^+^ T cells in draining cervical lymph nodes, spleen and blood revealed unaltered percentages in naive and transplanted Lewis rats. **B:** Percentages of 3.2.3^+^ cells in draining cervical lymph nodes, spleen and blood of Lewis wt rats, naive or 3 days post transplantation of L23 (+L23). In contrast to T cells the increase of 3.2.3^+^ cells indicated infiltration and expansion of NK cells and/or monocytes. **C:** Staining with mAb 3.2.3 of lymph nodes, spleen and blood of LEW.BH-NKC identified NK cells in naive rats or at different time points after transplantation of L23 cells. Detection of NK cells confirmed the increase in draining lymph nodes, spleen and blood at the early phase of immune response. The comparison of draining lymph node cells with population of mesenterial lymph nodes revealed a weak increase of NK cells. Results are shown as graphs summarising results of at least three independent experiments as mean ± standard deviation.

We assessed fraction of T cells in draining lymph nodes, spleen and blood of Lewis wt rats before and 3 days post transplantation to evaluate if a decrease in number of T cells is the cause of diminished proliferative activity in draining lymph nodes. However, proportion of T cells were unaffected by the graft **(** **Fig. 3A** **)**. In contrast, staining of the respective tissues of Lewis wt rats with mAb 3.2.3 showed an increase of 3.2.3^+^ cells after subcutaneous transfer of porcine cells with extended outcome in the blood **(** **Fig. 3B** **)**. Using congenic LEW.BH-NKC revealed that most of the 3.2.3^+^ cells in Lewis wt rats were presumably monocytes **(** **Fig. 3C** **)**. This conclusion was drawn of the drastically lower percentages of NKR-P1A^+^ cells in comparison to in 3.2.3^+^ cells in Lewis wt rats. Nevertheless, the increase of NK cells in all tested compartments was confirmed. Interestingly the observed raise of NK cells in the blood and spleen of LEW.BH-NKC was only detectable at day 3 after transplantation. On day 5 the percentages already dropped to basic levels or even below while in draining lymph nodes there was a constant even though slight increase of NK cells which could not be observed in mesenteric lymph nodes.

### NK Cells Reduce the Proliferative Response of Lymph Node Cells *in vitro*

Recently, we reported of a subset of human NK cells that inhibits IL-2 induced proliferation of bulk NK cells [17]. This subset represents only a small fraction of all NK cells. Therefore, the slight increase of NK cells in the draining lymph nodes might be sufficient to exhibit regulatory function on T cells. To prove the hypothesis that NK cells can generally interfere with T cell activation, naive lymph node cells were stimulated with L23 cells or fully MHC-disparate spleen cells from Lewis.1w rats in a mixed lymphocyte reaction (MLR). The stimulation of responder cells was performed in the absence or presence of NK cells derived from the spleen of the same individuals.

**Figure 4:**
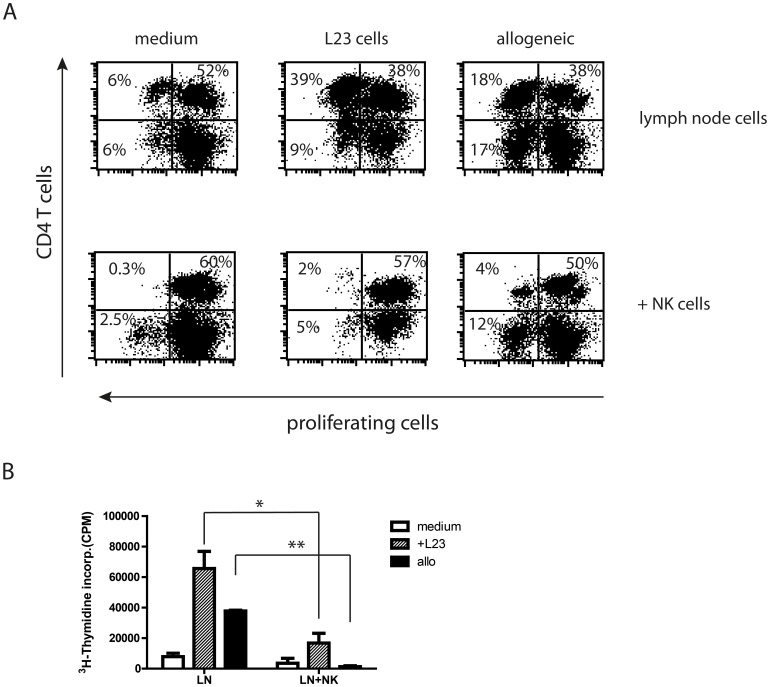
NK cells control the response of lymph node cells. A: Representative scatter gram of proliferating lymph node cells with or without supplement of autologes NK cells. 2×10^5^ lymph node cells from naive congenic LEW.BH-NKC were stimulated with 2×10^3^ porcine L23 cells or 2×10^5^ fully disparate spleen cells from Lew.1w rats in the absence or presence of 2×10^4^ negative isolated naive NK cells. LEW.BH-NKC cells were labelled with fluorescent dye CMFDA to follow proliferation in flow cytometry. **B:** Proliferation was also assessed by analysis of ^3^H thymidine incorporation to confirm flow cytometric results. The presence of NK cells drastically diminished the response of lymph node cells. Results shown as scatter grams represent a bulk of three independent experiments. Graph summarises results of at least three independent experiments as mean ± standard deviation. *p≤0.05; **p≤0.01.

Assessment of proliferation of lymph node cells was performed by flow cytometry using CMFDA-labelled lymphocytes and by ^3^H thymidine incorporation, respectively **(** **Fig. 4A and B** **)**. Both methods demonstrated significant reduction of proliferation of lymph node cells in the presence of NK cells. The inhibition was independent on the stimulus.

### NK Cells Control T Cell Response by Soluble Factors

**Figure 5:**
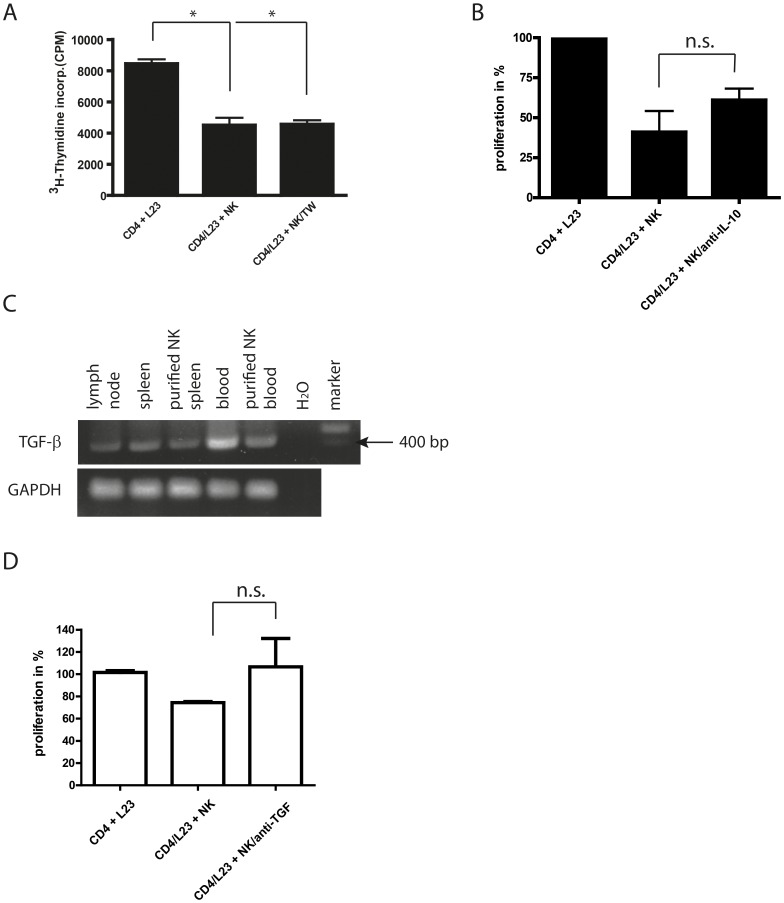
Control of CD4^+^ T cell proliferation by naïve NK cells is mediated by TGF-β. A: To evaluate the factors preventing proliferative T cell response, 2×10^5^ isolated CD4^+^ T cells as main responder to L23 stimulation (2×10^3^ cells) were incubated with 2×10^4^ NK cells directly or separated by a transwell (TW) from T cells and L23. The response of T cells was inhibited by NK cells independently on the possibility of contact formation. **B:** MLR with purified CD4^+^ T cells and NK cells in the presence of anti-IL-10 antibodies. IL-10 has known regulatory function and NK cells have been described to secrete IL-10. However, blockage of IL-10 with neutralising antibodies resulted only in a weak recovery of proliferation. Thus, IL-10 might play a role in regulation of T cells by NK cells but not basically. **C:** Assessment of TGF-β mRNA expression in bulk lymph node cells, splenocytes and of blood derived lymphocytes in comparison to purified NK cells. Lymphocytes were isolated from naive LEW.BH-NKC rats and subsequently prepared for mRNA isolation. The expression of TGF-β was compared to the house keeping gene GAPDH. TGF-β was detectable in bulk fractions and purified naive NK cells however relatively weak in lymph node cells of naive rats. **D:** Neutralisation of TGF-β using mAb in MLR of 2×10^5^ purified CD4^+^ T cells derived from LEW.BH-NKC rats in the presence of 2×10^4^ negative isolated NK cells. T cells were stimulated with 2×10^3^ L23 cells for 5 days. Although the inhibition of proliferation by NK cells was not significant in these experiments the addition of anti-TGF-β antibodies completely recovered the proliferation. Results presented as representative PCR analysis or as graphs summarising results of at least three independent experiments as mean ± standard deviation. *p≤0.05; n.s. = not significant.

The cytotoxic activity of NK cells against L23 cells (Fig. 2B) can be a feasible alternative as origin of less proliferation. A destruction of stimulator cells might decrease their capacity to induce proliferation of T cells. This possibility can be easily controlled by spatial separation of T cells/L23 cells and NK cells by transwells. These inserts allow the exchange of soluble mediators but avert contact dependent processes. We isolated CD4^+^ T cells to verify direct interaction between T cells and NK cells. Purified T cells responded with pronounced proliferation to stimulation with L23 cells. This proliferation could be inhibited by the addition of NK cells independently on the use of transwells **(** **Fig. 5A** **)**. These experiments give three indications: 1. The decrease of proliferation is not caused by NK cytotoxicity against the stimulators. 2. Soluble factors regulate the T cell response. 3. The control of T cells does not depend on accessory cells but is directly mediated between T and NK cells.

A well known cytokine affecting T cell regulation is IL-10 and its release has been reported in human and murine activated NK cells [18], [19], [20], [21]. Consequently, we added neutralising anti-IL-10 antibodies to MLR using purified CD4^+^ T cells as responder. However, this caused only a weak reversion of T cell proliferation which is a sign for additional agents with regulatory capacity **(** **Fig. 5B** **)**.

Another effective suppressive cytokine is transforming growth factor (TGF)-β. The assessment of mRNA level in the lymph nodes, spleen and blood of naive rats showed an expression in all tested compartments even though the lymph nodes seemed to express TGF-β in less distinct amounts in comparison to spleen and lymph nodes. In addition to bulk lymphocytes also purified naive NK cells expressed constitutively TGF-β **(** **Fig. 5C** **)**. NK cells revealed thereby a higher expression than lymph node cells. This indicates a possible function of TGF-β in T cell suppression. Thus, TGF-β was blocked by mAb. The supplement with anti-TGF-β antibodies to culture medium restored the proliferation completely **(** **Fig. 5D** **)**. Interestingly, we observed the reconstitution of proliferation even in the medium control which also showed signs of suppression after addition of NK cells (data not shown). Because of the complete prevention of T cell suppression by neutralising TGF-β antibodies this cytokine is most likely the main initiator of the observed inhibition.

### NK Cells Hold T Cells in a Steady State to Prevent Uncontrolled Response

**Figure 6:**
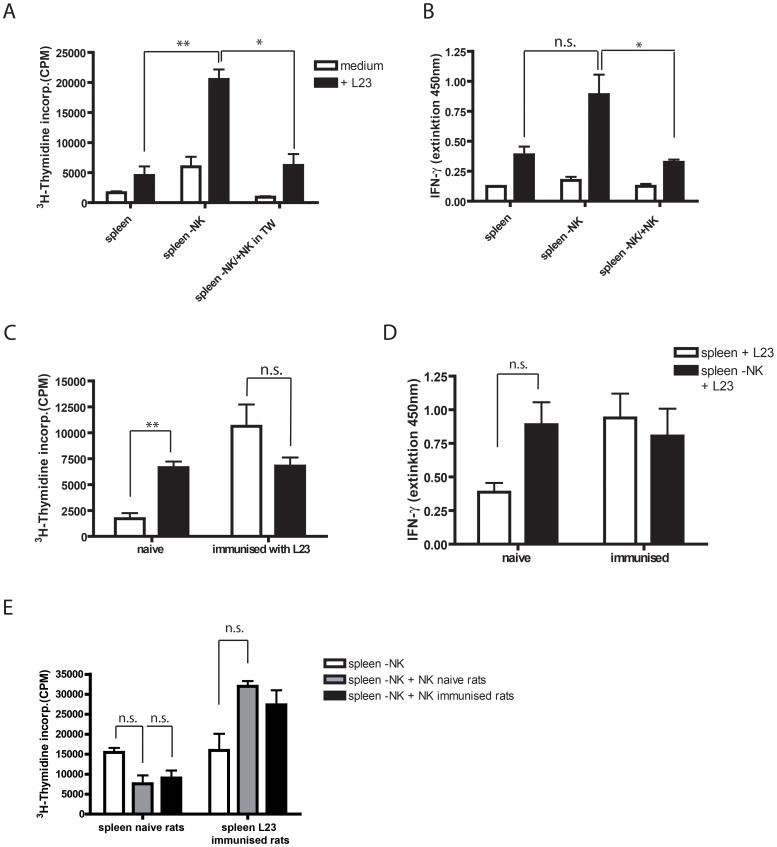
NK cells control the response of naïve but not of activated T cells. A: To confirm that NK cells rather control the T cell response than suppress, 2×10^5^ splenocytes were depleted of NK cells and response to 2×10^3^ L23 was assessed in the absence or presence of 2×10^4^ NK cells incubated in transwells. Depletion of NK cells increased the proliferative response of splenocytes significantly which could be reversed by contact independent return of NK cells in transwells (TW). **B:** The control of proliferation of T cells by NK cells in bulk splenocytes is combined with a control of cytokine response e.g. IFN-γ as one of the important inflammatory cytokines. **C:** For evaluation of regulatory function of NK cells at different stages of immune response, 2×10^5^ splenocytes from naïve and 14 days L23-immunised LEW.BH-NKC rats were used as responder cells. Splenocytes were incubated as bulk or NK depleted cells with 2×10^3^ L23 cells. Thus, for naïve cells this was the initial stimulation, for immunised splenocytes it was a specific re-stimulation. The naïve spleen cells responded to L23 stimulation with increased proliferation after NK-depletion whereas using immunised splenocytes a decrease in proliferation was detected. **D:** Assessment of IFN-γ revealed a strong increase in cytokine secretion in naïve splenocytes depleted of NK cells but a decrease in NK-depleted immunised splenocytes. **E:** To evaluate if NK cells derived from immunised rats lost the regulative capacity, 2×10^5^ spleen cells from naïve and immunised rats were depleted of NK cells, stimulated with 2×10^3^ L23 cells and cross-incubated with 2×10^4^ purified naive NK cells or derived from immunised rats. Stimulation of NK-depleted splenocytes with L23 cells induced comparable proliferation in spleen cells derived from immunised and rats. While the proliferation of naive splenocytes could be influenced by both NK cells isolated from naïve and immunised rats, no control of immunised splenocytes was detectable. In contrast, both type of NK cells increased the proliferation as could be observed in (C). Results are shown as representative scattergram of FACS analysis or as graphs summarize results of at least three independent experiments as mean ± standard deviation. **p≤0.01; *p≤0.05; n.s. = not significant.

The exposure of CD4 T cells to L23 cells leads to pronounced proliferation. Even in the presence of NK cells T cells start to proliferate however less distinct. This can be interpreted in two ways: 1. a necessary T cell response is suppressed which would help the pathogen or graft and therefore hard to believe and 2. NK cells prevent an overwhelming or redundant reaction of T cells but allow responses powerful enough to fight infection and foreign bodies. This would mean a control rather than suppression. To verify this hypothesis, we depleted NK cells from the spleen and stimulated splenocytes in presence or absence of NK cells (in transwells) with L23 cells. The depletion of NK cells from splenocytes increased spontaneous proliferation even by incubation simply in medium. The response increased further after stimulation with L23 cells **(** **Fig. 6A** **)**. Both, the medium control and the L23 simulated proliferation were significantly reduced after addition of NK cells to the culture. The increase of proliferation in NK-depleted splenocytes is associated with enhanced release of IFN-γ of these cultures which is also reversible by the addition of NK cells **(** **Fig. 6B** **)**. This indicates clearly a control of T cells in a steady state and in the induction of their reaction to an antigen.

### T Cell Proliferation after Establishment of Adaptive Immune Response cannot be Controlled by NK Cells

Yet, the results presented reflect the situation of the onset of an immune response against L23 cells. It has been reported that NK cells control ongoing adaptive immune response comparable regulatory T cells [19], [20]. Thus, we immunised LEW.BH-NKC rats with L23 cells intraperitoneal and followed the increasing titre of antibodies in the serum against L23 cells as a marker of full-blown adaptive immune response.

Antibodies were not detectable on day 3 and started to increase from day 5 after transfer of porcine cells and with saturation on day 14 after transplantation (data not shown). Thus, rats were sacrificed on day 14 after immunisation and restimulated with L23 cells in absence or presence of NK cells.

In contrast to naive rats the proliferation of immunised splenocytes decreased after depletion of NK cells **(** **Fig. 6C** **)**. This was in line with the release of IFN-γ which was also decreased even though not as pronounced as the proliferation **(** **Fig. 6D** **)**.

We thought of two possible explanations for the different outcome using L23-experienced splenocytes. First, there is a switch of NK cell function which would suggest a loss of regulative capacity of NK cells or second, activated T cells cannot be controlled even by naive NK cells. Therefore naïve and immunised splenocytes were depleted of NK cells and stimulated with L23 cells either alone or in the presence of naïve and immunised NK cells, respectively **(** **Fig. 6E** **)**. The results revealed a regulative function of both naive and immunised NK cells on naive T cells but not on experienced T cells. This gives an indication that T cells once fully activated cannot be influenced by NK cells.

## Discussion

In this study we describe the general capacity of NK cells to control T cell responses in rats. The regulation is based on the control of T cell responses in the initial phase of T cell activation. This is remarkable because of two findings: 1. the regulation is mediated by soluble factors but not (exclusively) by IL-10 and 2. NK cells do not have to be activated to exhibit regulatory capacity. This is in contrast to previous reports that found IL-10 mediated regulation of the immune response by NK cells after systemic or local infection [19], [20] in mice and in human using *in vitro* methods [18]. In these reports the activation of NK cells was the presumption for their regulative ability. Thus, the establishment of a barrier by naive NK cells that must be conquered for activation of T cells is a new aspect of NK cell biology.

In rats, assessment of NK cells is a double edged sword. More and more receptors are identified which enables a more detailed characterisation of rat NK cells, however the corresponding antibodies for detection of these receptors are not commercially available [22], [23]. In this study we introduce a congenic rat strain on Lewis background lacking the allele of NKR-P1B recognised by mAb 3.2.3 or 10/78. Thus, these congenic rats allow the clear identification and even more important the purification of NK cells without contamination of monocytes.

We used a xenogenic model because of the fast and complete rejection of cells which involve most likely NK cell participation. Porcine cells provoked also *in vitro* direct activation of NK cells. In regard of T cell proliferation L23 cells induced comparable reaction as complete MHC disparate allogenic Lewis/1w. We choose L23 cells as they can easily be transduced with the Luciferase gene to follow rejection of cells by luminescence imaging. Further on they have the characteristic to induce a response in T cells directly without bystander cells like host APC [16].

The rejection of L23 cells observed within five days after transfer indicates a predominant innate immune response. This is highlighted by the dramatic increase of 3.2.3^+^ cells in the blood of recipient Lewis wt rats. The transfer of L23 in congenic rats revealed that most of the detected 3.2.3^+^ cells in Lewis wt were not NK cells but presumably NKR-P1B^+^ monocytes. It has been shown that monocytes react very fast with increasing binding of mAb 3.2.3 after allogenic kidney transplantation [24] and this might explain the rigorous raise of 3.2.3^+^ cells in Lewis wt rats. Nevertheless, staining of lymphocyte composition in LEW.BH-NKC rats confirmed an increase of NK cells after cell transfer in the blood, spleen and as well in draining lymph nodes although little in the latter. Interestingly, the frequency of NK cells in spleen and blood rose until day 3 after transfer of cells and dropped to basic levels on day 5. In contrast, ongoing infiltration was detected in draining lymph nodes even on day 5 post transplantation. In addition, the number of T cells remained unmodified within the first 72 hours after transplantation in the draining lymph nodes as well as in blood and spleen which indicates non-established adaptive immune response.

On the other hand, assessment of proliferation in the draining lymph node revealed a drastic decrease of dividing cells after stimulation with L23 in a MLR. As source of this control we identified naive NK cells. Of note, the control by NK cells works directly on T cells without the help of accessory cells such as APC: Proliferation of highly purified CD4^+^ T cells stimulated in MLR was decreased in the presence of purified NK cells.

The reduced response in draining lymph nodes in this very early phase of the immune response was a surprise. A study injecting PHA subcutaneous in pigs reported of fast invasion of lymphocytes in the area of injection and increased numbers and proliferation of T cells in the draining lymph nodes within the first 48 hours [25]. Though, PHA is a very unspecific stimulator with activation of pathways independent of the CD3/28 complex thus not directly comparable to the model in this study. A more equivalent study using a murine colitis model showed a control of CD4^+^ T cells by NK cells *in vivo* [26]. Depletion of NK cells *in vivo* caused an uncontrolled T cell response yielding in elevated colitis scores. However, this control was dependent on contact formation and Perforin. In our model the application of transwells did not affect the regulative outcome on T cells and therefore soluble factors released by NK cells must be the agents of the control. A cytokine induction in NK cells without the presence of accessory cells is challenged [27] but could be shown in interaction with protozoan pathogens [28], [29] thus, is possible in general.

The detected activity of NK cells – probably in concert with monocytes – against the cell graft and the simultaneous delay of the onset of adaptive immunity might be a mechanism to protect the system against uncontrolled responses which can cause severe systemic complications. While the action taken against the porcine cells require an activation of NK cells, the invasion in the draining lymph node does not necessarily depend on activation [30]. It is important to note that the T cell response is not suppressed in terms of the ability to respond to an antigen but the establishment of a threshold by NK cells that T cells have to overcome thus only a strong or specific stimulus lead to a complete activation of T cells.

This theory is backed by the spontaneous (in the absence of any stimulation) increase of proliferation of splenocytes after depletion of NK cells which after stimulation with L23 cells led to a drastic increase of proliferation and cytokine release exemplary shown by IFN-γ secretion.

We tried to identify the cytokine released by NK cells responsible for the control of T cells. Numerous studies on regulatory T cells revealed IL-10 as an efficient suppressor cytokine (reviewed by [31]). The blockade of IL-10 with antibodies in our study however, revealed inconclusive results. Although a reverse of the control could be observed with neutralising antibodies the effect was weak and identification of IL-10 in NK cells was not convincing, too. While the NK cells of 50% of tested rats, congenic LEW.BH-NKC and Lewis wt, were completely negative of IL-10, in the other 50% we found IL-10 expression in a subset of naive NK cells (data not shown). On one hand this is very promising since regulatory function has been found only in a subset of bulk NK cells [17], [32], [33], on the other hand the effect of control of T cells by NK cells was highly reproducible in every tested rat. Of note, 3 days after cell transfer, IL-10 was absent in all tested NK cells even in those being IL-10^+^ in the naive state (data not shown). Nevertheless, NK cells derived from immunised rats were able to exhibit control function. From these results we conclude that IL-10 might be part of the control but regulatory function does not depend on this prominent cytokine.

In earlier studies it has been shown that murine NK cells can influence T cell activity with the help of TGF-β [34] and a contact dependent though probably cytokine mediated regulatory function could be found in human, too [21]. In this *in vitro* study induction of TGF-β and IL-10 expression in NK cells was described but functional outcome for T cell proliferation was no subject of this study. Therefore TGF-β is a good candidate. Indeed, blockage of TGF-β revealed a pronounced function in control of T cells. In addition, we detected TGF-β in naive purified NK cells and conclude therefore that TGF-β is the agent of the NK cell mediated control.

In order to confirm regulative activity in ongoing immune responses for the rat as it has been found in mice and in human, we used naive and immunised spleen cells. For immunisation we injected L23 intraperitoneal in LEW.BH-NKC rats and sacrificed rats 14 days thereafter when a high titre of antibodies against L23 in the serum indicated an established adaptive immune response. Incubation of NK cells derived from naive or immunised rats with naive splenocytes depleted of NK cells revealed control function for both types of NK cells which was completely abrogated when incubated with immunised splenocytes restimulated with L23 cells. These results are in line with the hypothesis that NK cells control the onset of T cell responses in the first place.

Taken together we showed in this study a TGF-β mediated T cell control by naive NK cells. This threshold prevents exceeded responses. This unexpected outcome points out again, that the role of NK cells is still incomplete understood.

## Methods

### Ethics Statement

All animal experiments were performed according to protocols approved by local Ethics Animal Review Board of the regional authorities for consumer protection and food safety of lower saxony (LAVES, Oldenburg, Germany) with the approval ID 08/1485 and 11/0489.

### Animals and Cell Culture

10–12 week old congenic LEW.BH-NKC rats (kindly provided by K. Wonigeit) were bred in the animal facility of the Medizinische Hochschule Hannover. The LEW.BH-NKC rats were generated by cross-breeding of Lewis rats with Black Hooded rats which lack the allele of NKR-P1B recognised by mAb 3.2.3. Rats were back-crossed with Lewis rats until the whole genome was identical with Lewis wt rats except the NKC. Rats were left naïve or immunised with 1×10^7^ L23 porcine B cells intraperitoneal. For initial experiments 10–12 week old Lewis wt rats were used (Charles River Laboratories, Sulzfeld, Germany). For allogeneic stimulation Lewis/1w rats were used (bred in the animal facility of the Medizinische Hochschule Hannover) which have the MHC haplotype uuu which is completely disparate to Lewis wt lll.

The Luciferase-transduced L23 cells [35] were maintained in RPMI/L-Glu/Pen-Strep medium supplemented with 10% fetal calf serum (FCS), 0.05 mM 2-mercaptoethanol, and 1 mM sodium pyruvate by continuous passages. In preparation of the mixed lymphocyte reaction L23 cells were harvested from the culture and allogeneic Lewis/1w cells were derived from the spleen. The cells were lethally x-rayed with 30 gray.

### Subcutaneous Transfer of Luciferase-transgenic L23 and Detection in IVIS

Culture derived L23 cells were washed three times in PBS. 5×10^5^ L23 cells were injected subcutaneous in the ear of Ketamin/Rompun anaesthetised rats. For evaluation of luminescence, 300 µg Luciferin were injected intraperitoneal 15 minutes after the placement of the cells under the skin (day 0) and after another 15 minutes rats were placed in an IVIS (IVIS 200 series, Caliper Life Science, Mainz, Germany). The anaesthesia was repeated on day 1 and 5 after transplantation. Luminescence was analysed using Living Image Software (Caliper Life Science, Mainz, Germany).

### Assessment of Cytotoxicity

Performed tests based on the JAM test described before [36]. In brief, L23 were incubated with 5 µCi [^3^H]thymidine for 24 hours. Cells were washed intensively and exposed in different effector to target ratios to purified NK cells for 4 hours. Culture was subsequently harvested and radioactivity was assessed after szintilisation using a β-counter (LKB Wallac). To analyse the degree of lysis of targets counts in cultures including NK cells were compared with control counts of [^3^H]thymidine labelled targets only which were set as 100%.

### Isolation/Depletion of Cell Population

Spleens were mashed through a strainer and erythrocytes were removed by a lysis buffer on NH_4_Cl basis. For NK cell depletion/positive selection spleen cells were incubated with biotinylated monoclonal antibody (mAb) against rat NKR-P1A/B (clone 3.2.3). Afterwards cells were washed and incubated with streptavidin-labelled micro beads (Miltenyi Biotec, Bergisch Gladbach, Germany) according to the manufactures instructions. NK cells were depleted of the spleen using a MACS MS column (Miltenyi Biotec). The flow-through (spleen/NK depleted) was gathered and the withholded NKR-P1A^+^ cells were collected after removing the column from the magnet. For negative selection of NK cells adherent monocytes and B cells were reduced in bulk spleen cells by incubation in culture plates for 4 h with subsequent incubation on nylon wool. Afterwards, cells were incubated on ice with a mixture of biotinylated mAb against rat CD3 (clone G4.18), CD4 (clone W3/25), CD8 (clone 3.4.1) and followed by incubation with streptavidin-labelled micro beads and micro beads conjugated polyclonal goat-anti-rat Ig antibodies (Miltenyi Biotec) to deplete remaining B cells. Again the isolation was performed using MACS MS columns.

#### Isolation of CD4^+^ T cells

Pooled mesenteric and cervical lymph node cells were stained with FITC-labelled mAb against rat CD4 (clone Ox-35; BectonDickinson, Heidelberg, Germany) and isolated by FACS using a FACSAria (BectonDickinson).

### Labelling of Lymph Node Cells with CMFDA

For assessment of proliferation in flow cytometry, pooled cervical and mesenteric lymph node cells were washed in serum-free medium and viable cells tracked through labelling with 4 µM CellTracker Green (5-chloro-methylfluorescin diacetate [CMFDA]; Molecular Probes, Eugene, Oregon, USA) for 20 min at 37°C. CMFDA is initially a non-fluorescent molecule able to pass the cell membrane and be cleaved in viable cells into the fluorescent, strongly hydrophilic form building a bulky water sheath averting its release through the intact cell membrane of viable cells. In dividing cells CMFDA is co-transmitted with the cytosol from the mother to the daughter cells which results in half fluorescence intensity. Lymphocytes were subsequently washed in RPMI medium warmed to room temperature (20°C) containing 10% human serum and were further incubated for 30 min at 37°C. After a single final wash labelled lymphocytes were incubated in MLR.

### Mixed Lymphocyte Reaction

2×10^5^ lymph node cells, splenocytes - untreated or depleted of NK cells - and isolated CD4^+^ T cells were stimulated with 2×10^3^ lethally irradiated L23 cells or 2×10^5^ allogeneic splenocytes derived from Lewis 1w rats in 96 well-round bottom plates for 5 days and pulsed with 0.5–1 µCi [^3^H]thymidine/well for 16 hours, respectively. The MLR was variegated by adding 2×10^4^ isolated NK cells either directly to the responder cells or separated by cell culture inserts (Nunc, Roskilde, Denmark). In other experiments 2.5 µg anti IL-10 (BD Bioscience, Heidelberg, Germany) and anti-TGF-β (R&D Systems, Wiesbaden, Germany) per well were added, respectively. [^3^H]thymidine incorporation was assessed after szintilisation using a β-counter (LKB Wallac, Turku, Finland).

IFN-γ production was measured in the supernatants of the culture by enzyme-linked immunoabsorbant assay (ELISA).

### Assessment of mRNA Expression

Naive bulk lymphocytes derived from lymph nodes, spleen and blood and negative isolated NK cells from spleen and blood were lysed and mRNA was isolated using NucleoSpin RNA II kit (Macherey-Nagel, Düren, Germany) according to manufactors instructions. mRNA was subsequently translated in complementary DNA by supplementing 1 µg RNA with Oligo(dT) primer and RevertAid Transcriptase (both Fermentas, St. Leon-Rot, Germany) and incubation for 1 h at 42°C. Reaction was stopped at 72°C for 10 min. DNA was amplified by PCR using primer for TGF-β: forward CCG CAA CAA CGC AAT CTA and reverse TGA GGA GCA GGA AGG GTC. As house keeping gene GAPDH was amplified by forward CCT TCA TTG ACC TCA ACT ACA TG and reverse CTT CTC CAT GGT GGT GAA GAC. PCR included 30 cycles with 30 sec 94°C, 30 sec 58°C and 1 min 30 sec 72°C.

### Statistical Analysis

Results are presented as the mean ± standard deviation. Each experiment was performed independently three to four times. Statistical analysis was generally performed with the unpaired Student’s *t*-test using prism software (Graph Pad Software, San Diego, CA, USA).
